# Applying the Triangle Taste Test to Assess Differences between Low Sodium Salts and Common Salt: Evidence from Peru

**DOI:** 10.1371/journal.pone.0134700

**Published:** 2015-07-30

**Authors:** Lorena Saavedra-Garcia, Antonio Bernabe-Ortiz, Robert H. Gilman, Francisco Diez-Canseco, María Kathia Cárdenas, Katherine A. Sacksteder, J. Jaime Miranda

**Affiliations:** 1 CRONICAS Center of Excellence in Chronic Diseases, Universidad Peruana Cayetano Heredia, Lima, Peru; 2 School of Public Health and Administration, Universidad Peruana Cayetano Heredia, Lima, Peru; 3 Department of International Health, Johns Hopkins Bloomberg School of Public Health, Johns Hopkins University, Baltimore, Maryland, United States of America; 4 Área de Investigación y Desarrollo, Asociación Benéfica PRISMA, Lima, Peru; 5 Department of Medicine, School of Medicine, Universidad Peruana Cayetano Heredia, Lima, Peru; German Institute of Human Nutrition Potsdam-Rehbruecke, GERMANY

## Abstract

**Background:**

In resourced-constrained settings, daily cooking practices are still the norm. Replacing sodium in regular salt to produce potassium-enriched salts are potential alternative routes to reduce sodium intake, paired with the benefit associated with potassium intake. This change would likely have effects on palatability and taste of prepared foods, yet a threshold to discriminate sensorial changes can be determined. The main goal of this study was to assess if the use of potassium-enriched salt substitutes lead to perceived differences in taste utilizing a sensory discrimination test.

**Methods and Results:**

A triangle taste test was conducted and participants were offered samples of cooked rice prepared with different salts. The only ingredient that differed in the preparation was the salt used: 100%NaCl (regular salt) and salts where sodium was replaced by 50%, 33% or 25% KCl (potassium-enriched salt). Comparisons were carried out according to the minimum number of correct judgments. A total of 156 subjects, 49% males, mean age 41.0 years (SD±15.5) years, participated in the study. Samples using 25% potassium-enrichment were indistinguishable in terms of taste from regular salt, whereas samples with 33% and 50% potassium-enrichment were distinguishable. Results were consistent when stratified by sex and age. Less than 10% of participants attributed the differences to bitterness or metallic flavor.

**Conclusions:**

The 25% potassium-enriched salt is indistinguishable from regular salt. These findings suggest a potential to achieve sodium intake reduction strategies in cooking practices by substituting regular salt with potassium-enriched salt without affecting palatability.

## Introduction

Worldwide, hypertension is considered a major risk factor for cardiovascular disease, especially heart attack and stroke [1]. A recent analysis of regional and global trends of systolic blood pressure found that though there was a decreasing trend observed globally since 1980, the decline per decade was higher in high-income settings (Australasia, North America and Western Europe), it actually rose in other regions, and systolic blood pressure is currently highest in low-income and middle-income countries [2]. In these resource-constrained settings there are few strategies to maintain blood pressure within appropriate ranges, reflecting the challenge of affordable implementation in health care systems.

Increased sodium consumption is associated with increased blood pressure, whereas lower sodium intake does decrease blood pressure in adults [3–7]. Therefore, one of the strategies largely advocated worldwide to reduce the burden of high blood pressure emphasizes the implementation of salt reduction strategies [8–10]. Additionally, low potassium intake is associated with an increased risk of hypertension, and a high ratio of sodium/potassium intake has been proposed as a potent risk factor for hypertension and cardiovascular disease than each factor alone [11, 12]. Thus, the increase of dietary potassium might reduce mean systolic and diastolic blood pressure levels, and could also contribute to the prevention of hypertension, especially in populations with elevated blood pressure [13, 14].

The most recent WHO guidelines strongly recommend a reduction to less than 5 grams per day among adults with or without hypertension [15]. Yet, the successful implementation of this recommendation, especially in developing countries where day-to-day food preparation and cooking practices are substantial, might be difficult to achieve without taking into account the role of salt on palatability of foods associated with flavor and taste [16, 17].

Replacing the sodium in the regular salt to produce potassium-enriched salts are potential alternative routes to reduce sodium intake, paired with the increased benefit associated with higher potassium intake. However, increasing potassium in the salt used for food cooking could introduce noticeable changes in flavor and/or palatability such as saltiness, bitterness, or metallic taste [18], with potential impact on consumer liking [19]. The main goal of this study was to assess if the use of potassium-enriched salt substitutes lead to perceived differences in taste utilizing a triangle taste test, a type of sensory discrimination test. This technique is employed to determine if a sensory difference between two products can be detected by consumers; for instance, assuming participants cannot perceive a different taste, then liking will not be affected.

## Materials and Methods

### Study design

A cross-sectional study using a triangle taste test technique, utilized in sensory analysis, was conducted to evaluate whether using salts containing varying proportions of sodium chloride (NaCl) and potassium chloride (KCl) in rice preparation could be detected.

### Setting and participants

This study was part of the initial exploratory phase of a larger pragmatic randomized community-based intervention to introduce a low-sodium potassium-enriched salt substitute to reduce blood pressure at the population level (ClinicalTrials.gov number: NCT01960972). The protocol of this umbrella study has been reported and is available elsewhere [20].

Participants from the six villages of Tumbes to be enrolled in the larger umbrella study were randomly selected from 2010 census, the most updated available. Males or females aged 18 years and over, capable of understanding study procedures, capable of providing informed consent, and who were full-time residents in the area were considered eligible.

### Procedures

For the triangle taste test, regular salt (100% NaCl) and potassium-enriched alternative salts were used in the preparation of rice, Peru’s most common staple food. In Peru, we had access to regular salt and a 50% NaCl/50% KCl potassium-enriched salt, both containing small amounts of iodine as per local regulations following international standards [21]. By combining appropriate proportions of these two salts, we generated salts containing 33% and 25% KCl concentration. Thus, in addition to regular salt, three types of potassium-enriched salts were used in the preparations: (i) 50% NaCl + 50% KCl, (ii) 67% NaCl + 33% KCl, and (iii) 75% NaCl + 25% KCl.

A single trained person did all the preparations using standardized techniques. To ensure consistency in food preparation, a simple formula yielded for 10 portions was cooked with the following ingredients: rice (150 grams), salt (5 grams), and water (400 grams). All the ingredients were combined in a pot and then boiled for a period of 30 minutes. At the end of this period, heat was turned off and rice was let cool for 5 minutes. Finally, rice was served in plastic recipients. In this way, the only element that differed in the food preparation was the type of salt used and, in doing so; it was expected to obtain rice preparations that might differ only on taste (unobserved) but were otherwise similar across a range of other factors, such as appearance, size, color and smell. No additional seasonings were utilized in the process.

### Experiment

**Fig 1:**
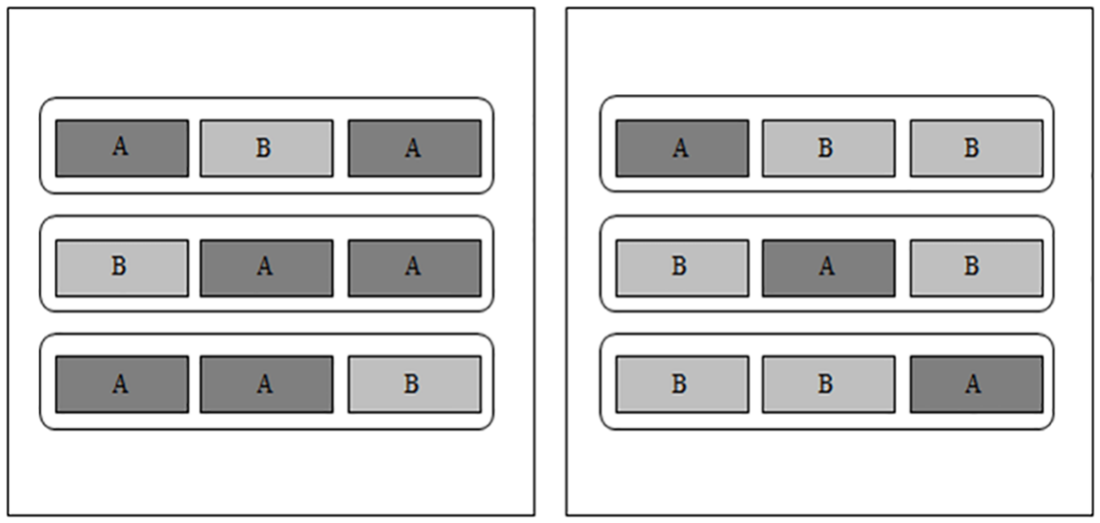
Sample distribution. Example 1. Example 2. Participants received in each set two identical samples (dark grey) and one odd sample (light grey): Example 1: ABA, BAA, and AAB. Example 2: ABB, BAB, and BBA.

We were interested if participants were able to identify the odd sample when exposed and after eating a set of three food samples cooked with different ratio of sodium/potassium salts. All samples were coded with a three-digit random number and participants were presented to three sets of food. Each set had two identical samples and one odd sample, and they were presented simultaneously in a predetermined counterbalanced order. The samples were randomly presented to avoid positional bias since the middle sample is usually chosen as odd. Possible combinations of samples were: AAB, ABA, BAA, BBA, BAB, and ABB. An example of the distribution of sets and samples used, including the variation of potassium-enriched concentration, is shown in Fig 1.

The ingredients of the food samples and the experiment procedures were explained to the participants and they were asked to identify the odd sample. If the participant was unable to determine which sample was different, they were asked to guess. Between tasting samples and sets, a glass of water was offered to the participants to avoid sensory fatigue [22].

After completing the experiment, participants were asked to complete a short questionnaire to identify the sample they believed to be the odd one as well as to indicate the degree of difference between samples (slight, moderate, or high). At the end of the experiment, demographic data, including sex, age, education level, marital status, and currently working status, was also collected.

### Sample size

With a power over 90% at the 5% level of significance, the triangle taste test was planned to include146 participants. As the probability of correctly respond by chance is 33% in a triangle taste test, this sample size provided us with enough power to state that there is not a detectable difference between different samples [23]. In addition, at least 53 participants in each subgroup were needed to guarantee a power of at least 80% to state that there is not a detectable difference between different samples in the subgroup analysis [24].

### Statistical analysis

After double data entry, the information was transferred to STATA 11 (STATA Corp, College Station TX, US) for analysis. Initially, description of demographic characteristics of participants was shown by using proportions.

To determine if the total number of correct responses for the total number of participants was statistically significant, we used the critical number of correct responses in a triangle taste test. Thus, we computed a chi-squared based on the following formula:

$$X^{2}=\frac{{\left(|4a-2f|-3\right)}^{2}}{8n}$$

In this formula, “a” is the proportion of participants who answered correctly, “f” is the proportion of people who did not answer correctly, and “n” is the number of participants. Results were also performed stratified by sex, age and education level. Additional comparisons were performed using Chi-squared test or Fisher exact test as needed.

### Ethical issues

Oral informed consent was obtained from all participants before enrollment. Oral consent was used due to low rates of literacy in the study setting, and the interviewer signed the form to document participant’s consent. This project and its procedures were reviewed and approved by Institutional Review Boards (IRBs) at Universidad Peruana Cayetano Heredia (Peru) and Johns Hopkins Bloomberg School of Public Health (US).

## Results

### Baseline characteristics

A total of 156 participants, 77 (49%) were males, mean age 41.0 (SD15.5) years, were enrolled in the study. Characteristics of the study population are detailed in the Table 1.

**Table 1:** Demographic characteristics of the study population.

Variables	N (%)
** Sex**	
Female	79 (50.6%)
Male	77 (49.4%)
** Age**	
< 40 years	81 (51.9%)
≥ 40 years	75 (48.1%)
** Education level**	
< 12 years	129 (82.7%)
≥ 12 years	27 (17.3%)
** Civil status**	
Never married	42 (26.9%)
Ever married	114 (73.1%)
** Currently working**	
No	86 (55.1%)
Yes	70 (44.9%)

### Correct identification of the odd sample

Samples using 25% potassium-enrichment were indistinguishable from regular salt. Participants were able to distinguish the odd sample in preparations that contained 50% and 33% of KCl (Table 2).

**Table 2:** Results of the triangle taste test: Detection of difference in rice flavor affected by partial sodium chloride replacement^a^.

	Number of judgments for rice flavor with replacement of NaCl by						
		Sex		Age		Years of education	
Replacement level (%)^b^	Total	Males	Females	< 40 years	≥ 40 years	< 12 years	≥ 12 years
		(n = 77)	(n = 79)	(n = 81)	(n = 75)	(n = 129)	(n = 27)
50% KCl	82/156***	44/77***	38/79*	43/81***	39/75**	64/129***	18/27**
33% KCl	69/156*	38/77*	31/79	36/81*	33/75	60/129**	9/27
25% KCl	63/156	32/77	31/79	31/81	32/75	54/129	9/27

^a^ Number of judgments correct divided by total in triangle tastes.

^b^ The proportion of sodium of the reference rice replaced by equivalent amounts of the potassium salt. Significant level:

* = significant at P = 0.05,

** = significant at p = 0.01,

*** = significant at p = 0.001

When the analyses were stratified by sex, age, and education level, the result did not differ markedly from the main findings: participants were not able to distinguish samples with 25% KCl; however, the odd sample was distinguishable when salt with 50% KCl (Table 3). Of note, females and those aged 40 years and above were not able to appropriately distinguish samples prepared with 33% KCl. Although the same trend was observable for those with ≥12 years of education, power to detect differences can be an issue in this case.

**Table 3:** Results of the triangle taste test: Reported differences according to participants’ responses.

	50% NaCl + 50% KCl			67% NaCl + 33% KCl			75% NaCl + 25% KCl		
	Incorrect	Correct	p-value*	Incorrect	Correct	p-value*	Incorrect	Correct	p-value*
**Reported difference (%)**									
Slightly	33.8%	40.2%	0.58	39.1%	42.0%	0.26	37.6%	17.5%	0.03
Moderate	56.8%	53.7%		47.1%	52.2%		52.7%	69.8%	
High	9.4%	6.1%		13.8%	5.8%		9.7%	12.7%	
**Reported taste (%)**									
Bitter taste	4.1%	2.4%	0.58	0.0%	4.4%	0.05	2.2%	3.2%	0.69
Metallic taste	10.8%	7.3%	0.45	8.1%	10.1%	0.65	12.9%	9.5%	0.52

*P-values were calculated using Chi-squared tests.

### Difference and reported taste of the odd sample

The degree of difference between samples that most participants identified was moderate (Table 3). In addition, when asked about the flavor of the reported odd sample tasted very few acknowledged them as bitter or metallic taste. There was no evidence of a difference in reported taste between those who did and did not identify correctly the odd sample.

## Discussion

### Main findings

Palatability is one of the main concerns, if not the main one, when considering substituting NaCl present in salt by KCl. The findings of this study indicate that changes in taste by using a 75%NaCl/25%KCl salt substitute were not discernible when compared to common salt. These results were consistent even when the analysis was performed stratified by sex and age implying that palatability was similar between products independently of sex and age. Moreover, these sub-group analyses show that especially women and those aged 40 years and above were not able to discern samples with 33% KCl. Henceforth, replacing sodium for potassium in daily cooking practices can be a potential avenue to introduce prevention initiatives aimed at improving cardiovascular health.

It is possible to replace NaCl with KCl in food preparations, because of both its saltiness and its comparable physical properties [25]. Both salts are colorless crystals and soluble in water, but unlike sodium, potassium has not been associated with progression towards hypertension and subsequent cardiovascular diseases [26]. The two salts could be used in a salt substitute either alone or in combination, though some may find KCl to have a bitter, chemical, or metallic taste [18]. The excess of potassium in diet is not a common problem: hyperkalemia by potassium intake would be seen with large infusions or oral doses of several grams of potassium chloride. However, in patients with severe and terminal chronic kidney disease, as well as those using some medication, accumulation of potassium can cause hyperkalemia. Thus, the safety profile of the salt substitute will be guaranteed, because the changes will be relatively minor compared to the levels when potassium does produce some negative health effects. Based on our study, although a small proportion of participants detected some bitterness or metallic taste, using an appropriate combination of KCl and NaCl appears to minimize these unpleasant potassium-related tastes.

### Comparisons with other studies

One of the main barriers in reducing salt intake is changes in palatability. However some studies have shown that it is possible to reduce gradually the amount of NaCl in the main sources of salt without affecting taste, such as the case of low-salt bread [27, 28]; but there is an additional benefit obtained when potassium is added, as previously detailed.

Our results agree with previous studies in that the greater the concentration of KCl in the substitute, the greater chance that it will be distinguishable in sensory discrimination tests. For example, several years ago, Salovaara showed it was possible to reduce the salt content of bread in about one third with no significantly detectable disadvantage in bread taste [29]. In addition, this report found that potassium chloride may replace 20% of the sodium equivalent normally added to bread without causing difference in flavor. Recently, Li et al compared a salt substitute with 65% NaCl, 25% KCl, and 10% magnesium sulfate to common salt. Changes in saltiness, flavor and acceptability of both home-cooked foods and a standard salt-seasoned soup were not different between study groups [30]. Another study replaced a fraction of the NaCl in white bread with KCl, and found that up to 30% of the NaCl could be replaced by KCl and have similar acceptability scores to the standard white bread [31].

Some data suggests that salt preference can be learned [30]. In this regard, it is possible that among participants with more frequent exposure to KCl, their concentration threshold of detection of KCl gradually decreased over time. This, in turn, provides an additional major opportunity to transit, gradually, towards higher NaCl substitution practices that contribute to accrue longer-term health-related benefits. Nevertheless, information regarding the effectiveness of changes in salt preference due to gradually reduction of salt is scarce [32].

### Public health impact

The potential expected benefit of the use of potassium-enriched salt at the population level lies on its demonstrated effects to reduce blood pressure through two different mechanisms: moderately reducing sodium intake and increasing potassium in the diet. One US-based study enrolled people with mildly elevated blood pressure and replaced their common salt with Smart Salt (50% NaCl, 25% KCl, and 25% magnesium ammonium KCl hydrate) over a period of eight weeks, which led to a significant reduction in systolic blood pressure [33]. A potassium-enriched salt substitute might have also a benefit effect on normotensive subjects: for example, a randomized controlled trial in a rural Chinese population showed an overall difference in systolic and diastolic blood pressure of 2 mmHg between two groups consuming either the salt substitute or normal salt after 24 months of follow-up [34]. Finally, in a study of five kitchens of a veteran retirement home in which groups received either potassium-enriched salt (experimental group) or regular salt (control group), the experimental group had better cardiovascular disease survivorship than the control group [35]. Moreover, according to our findings, it is possible to implement salt substitutes containing 33% KCl in some sub-groups of the population. Reasons for reported differences need to be further studied.

Based on this data, potassium-enriched salt substitutes may be a feasible approach to reducing sodium intake at the population level. The survey presented here was performed as part of the exploratory phase of a research study with the goal of implementing a potassium-enrichedsalt substitute to reduce blood pressure at the population level. Because randomly-selected people in this Peruvian population were not able to differentiate between a salt substitute with 25% and common salt, there is a good possibility that this implementation can be successful using the 25% potassium-enriched salt as a starting point for its implementation.

### Strength and limitations

The main strengths of this study included the use of a random sample of participants to evaluate different concentrations of KCl in the substitutes, including the random presentation of samples. However, this study has also some limitations. First, we only used rice in our experiments as we needed to standardize techniques including cooking procedures. Further studies are suggested to assess the potential impact of salt substitutes in food preparations. Second, some participants could have achieved sensory fatigue because of repeated assessments despite of using a glass of water to reduce this trouble. Finally, we could not find differences in comparisons according to demographic variables because of low number of participants in subgroups, especially in the case of education level (≥12 years of education). However, there is a clear trend in correct responses when the concentration of potassium in the sample is decreasing.

### Conclusions

Samples using 25% potassium-enriched salt was indistinguishable from regular salt. These findings suggest a potential to achieve sodium intake reduction strategies in cooking practices by substituting regular salt with potassium-enriched salt without affecting palatability.
